# Functional reconstruction of elbow flexion with latissimus dorsi muscle rotational transfer: two case reports

**DOI:** 10.1186/s13256-023-04178-4

**Published:** 2023-10-31

**Authors:** Yuka Kobayashi, Yoshiyasu Uchiyama, Shinji Yoshida, Ikuo Saito, Takayuki Ishii, Daisuke Nakajima, Shou Yanagisawa, Masahiko Watanabe

**Affiliations:** 1 Department of Orthopaedic Surgery, Tokai University Hachioji Hospital, Ishikawacho, Hachioji, Tokyo 192-0032 Japan; 2 Department of Orthopaedic Surgery, Surgical Science, Tokai University School of Medicine, Kanagawa, 259‑1193 Japan; 3 Department of Orthopaedic Surgery, Isehara Kyodo Hospital, Kanagawa, 259-1187 Japan

**Keywords:** Biceps brachii, Brachialis, Case report, Elbow flexion, Latissimus dorsi muscle flap, Pedicled flap

## Abstract

**Background:**

We report two cases of biceps brachii and brachialis paralysis due to musculocutaneous nerve injury in which elbow joint flexion was reconstructed using rotational transfer of the latissimus dorsi muscle with sutures to the radial and ulnar tuberosities, thereby enabling flexion by simultaneous activation of the humeroradial and humeroulnar joints. In cases of associated brachialis paralysis, weaker flexion strength can be expected when the forearm is in a pronated position than when it is in a supinated state. To the best of our knowledge, no previous study has reported the rotational position of the forearm during elbow joint flexion reconstruction.

**Case presentation:**

Case 1 involved a 30-year-old Asian male who presented with a rupture of the musculocutaneous, median, radial, and ulnar nerves. Reconstruction was performed by rotational transfer of the latissimus dorsi muscle. In this case, the supination and pronation flexion forces were equal. Case 2 involved a 50-year-old Asian man who presented with partial loss of the musculocutaneous nerve, biceps brachii, and pectoralis major due to debridement. Reconstruction was performed by rotational transfer of the latissimus dorsi muscle. In this case, supination and pronation flexion strengths were demonstrated to be equal. Our reconstruction method used the rotational transfer of the latissimus dorsi muscle; the distal muscle flap was divided into radial and ulnar sides to allow elbow joint flexion by simultaneously activating the humeroradial and humeroulnar joints. These sides were then fixed to the anchors at the radial and ulnar tuberosities. Finally, they were wrapped around the myotendinous junction of the biceps brachii or brachialis and secured using sutures.

**Conclusions:**

Although larger studies are required to verify these methods, this case study successfully demonstrates the following: (1) the flexion strength in the supinated position was equal to that in the pronated position; (2) the stability of the humeroradial and humeroulnar joints was unaffected by the forearm's rotational position; and (3) a satisfactory range of motion of the elbow joint was obtained, with no complications.

## Introduction

The biceps brachii and brachialis are the critical muscles involved in elbow flexion. Specifically, the biceps brachii are innervated by musculocutaneous nerves. In contrast, the brachialis is innervated by the musculocutaneous nerve at the superficial head and the radial nerve at the deep head [1, 2]. The superficial head is larger than the deep head, originates from the humerus, and inserts into the ulna. Thus, the musculocutaneous nerves are integral to elbow flexion. The mechanical moment of the biceps brachii is high when elbow flexion is performed with the forearm in the supinated position. Furthermore, the brachialis is responsible for elbow flexion, regardless of the forearm’s rotational position. The treatment depends on whether the cause of the injury is a rupture of the distal biceps brachii muscle or musculocutaneous nerve damage, resulting in paralysis of the biceps brachii and brachial muscles. When the biceps brachii and brachialis are paralyzed owing to musculocutaneous nerve injury, the forearm cannot flex the elbow joint in the pronated or supinated position.

In distal biceps brachii rupture cases alone,soft tissue reconstruction and functional restoration using an anterolateral thigh flap have been previously reported [3] but are not indicated for patients with paralysis. Moreover, no specific guidelines exist for elbow joint flexion reconstruction, as it depends on the etiology and diagnosis of each injury [4]. However, there are various grafting operations to restore active elbow flexion, including Steindler flexoplasty [5, 6], triceps brachii transfer [7], pectoralis major transfer [8], transfer of free muscles such as the gracilis [9, 10], and latissimus dorsi muscle bipolar rotational transfer [11–14]. Specifically, the Steindler method is not indicated in cases of decreased forearm flexor muscle function, and the triceps brachii transfer is a reconstruction method performed by replacing the extension function of the elbow joint; however, this is not indicated in cases involving radial nerve paralysis. Furthermore, pectoralis major transfer is not widely used because of its poor cosmetic outcomes. Free muscle transfer yields favorable flexion strength, although there is no protective sensation, and muscle atrophy is more likely to occur after this procedure than after pedicle transfer. Alternatively, latissimus dorsi muscle rotational transfer includes elbow flexion reconstruction and enables single-stage soft tissue reconstruction. Thus, this method was selected for patients who presented with paralysis of the biceps brachii and brachialis resulting from musculocutaneous nerve injury and soft tissue damage accompanied by loss of elbow flexion.

Reconstruction via latissimus dorsi muscle rotational transfer is commonly performed by suturing a muscle flap to the radial tuberosity where the biceps brachii is inserted [11–14]. However, the radial tuberosity rotates posteriorly upon forearm pronation, and the attached muscle flap is pulled between the radius and ulna. In daily life, the elbow joint must be flexed with a strong force by pronation of the forearm during movements such as grabbing an object and pulling it close to the chest or face. In cases of associated brachialis paralysis, weaker flexion strength can be expected when the forearm is in a pronated position than when it is in a supinated state. However, to the best of our knowledge, no previous studies have described the rotational position of the forearm during elbow joint flexion reconstruction.

Herein, we present two cases of biceps brachii and brachialis paralysis due to musculocutaneous nerve injury and reconstruct elbow joint flexion using rotational transfer of the latissimus dorsi muscle with sutures to the radial and ulnar tuberosities, thereby enabling flexion by simultaneous activation of the humeroradial and humeroulnar joints.

## Case presentations

### Case 1

**Fig. 1:**
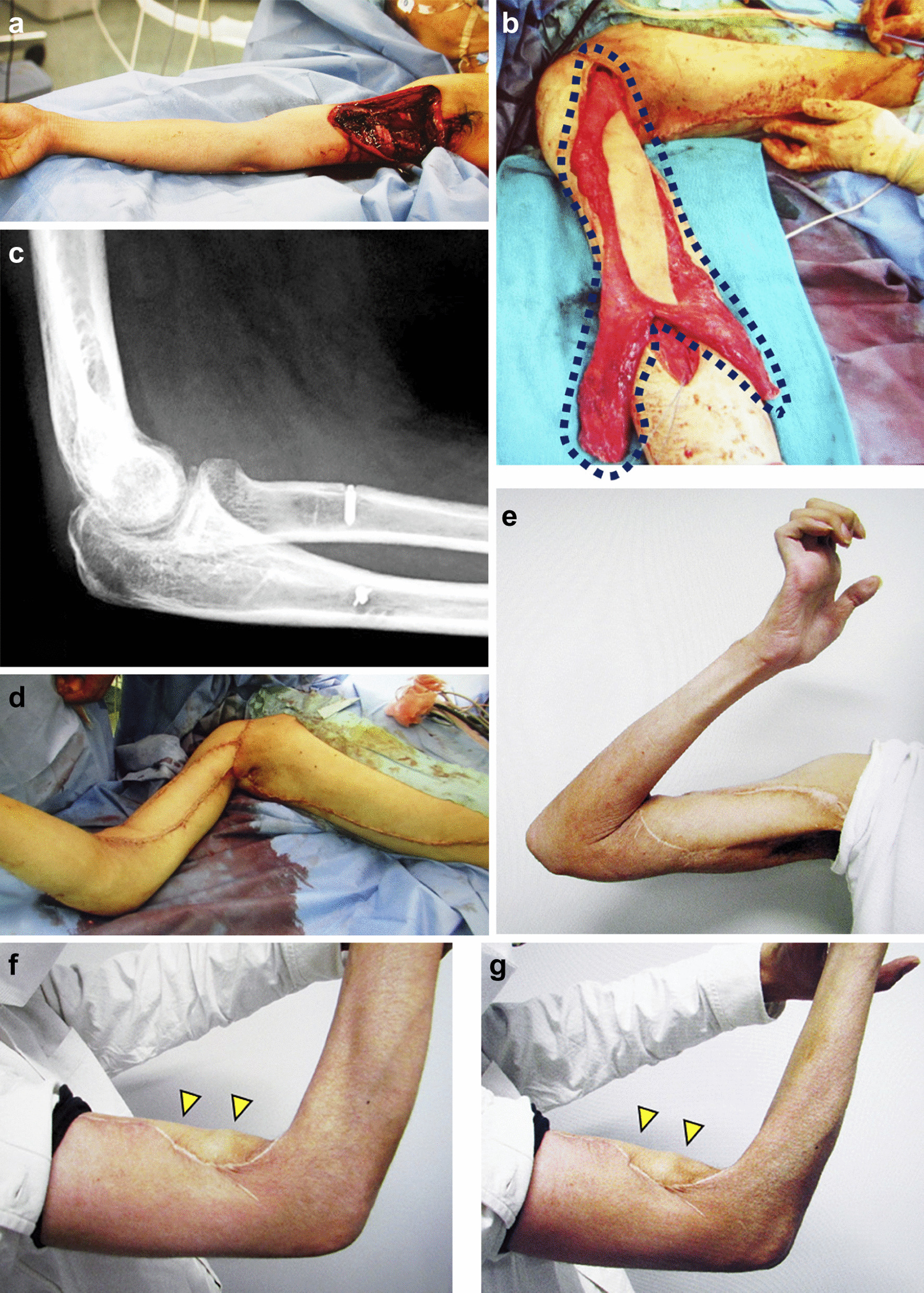
Case 1. **a** There was severe damage to the musculocutaneous nerve, and a primary suture or graft was deemed impossible. **b** The distal muscle flap of the transferred llatissimus dorsi muscle was divided into two. **c** Simple X-ray imaging shows fixation to the radial and ulnar tuberosities with GII^®^ anchors. **d** Elbow flexion and soft tissue were simultaneously reconstructed with a latissimus dorsi musculocutaneous flap. **e** Elbow’s active range of motion was 10–145°. **f** Forearm pronation was performed with extension resistance. Active contraction of the latissimus dorsi muscle with elbow flexion was confirmed (Yellow triangles indicate latissimus dorsi muscle). **g** Active contraction of the latissimus dorsi muscle was confirmed similarly to forearm supination (Yellow triangles indicate latissimus dorsi muscle)

Case 1 was that of a 30-year-old Asian male who injured his right proximal upper arm while breaking a glass door. The injury created deep lacerations in the right upper arm, disrupting the musculocutaneous, median, radial, and ulnar nerves. The brachial artery and vein severing and rupture of the musculocutaneous, median, radial, and ulnar nerves were observed (Fig. 1a). Furthermore, there was severe damage to the musculocutaneous and radial nerves, and on the day of the injury, a primary suture was deemed impossible. However, the radial nerve was repaired using a sural nerve cable graft on the injury day. Moreover, the severity of the injuries to the musculocutaneous nerve and biceps brachii made grafting impossible. Radiographs showed no fractures, and the primary diagnosis was paralysis of the right upper arm due to the rupture of the musculocutaneous, median, radial, and ulnar nerves. Paralysis of the biceps brachii and brachialis due to musculocutaneous nerve injury resulted in the inability to perform elbow flexion. Thus, four weeks after the injuries were sustained, a bipolar latissimus dorsi muscle with a skin paddle was used to restore elbow joint flexion and reconstruct the soft tissue. For bipolar transfer, the muscle flap was fixed to the coracoid process using a 4.5 mm HEALIX ADVANCE BR anchor (DePuy Mitek, Raynham, MA, USA). Thus, the muscle belly of the distal latissimus dorsi was separated into two pieces that were fixed to the radial and ulnar tuberosities using GII anchors (DePuy Mitek) (Fig. 1b, c), and muscle tension was adjusted to ensure that the elbow spontaneously remained at 90° at the end of fixation (Fig. 1d). Furthermore, the elbow joint was externally splinted to maintain a 90° bend with the forearm in full supination for six weeks following the operation. Physiotherapy was initiated seven weeks postoperatively, and the patient began working on active and passive flexion of the elbow joint. As the case was complicated by triceps brachii paralysis, the elbow extension was increased by 30° every two weeks. Three months postoperatively, the active range of the elbow recovered to 25–135°. However, the lower median and ulnar nerve paralysis persisted for 4 years after surgery. Notably, the elbow’s active range of motion was 10–145° four years postoperatively, and the British Medical Research Council grade (MRC) assigned for elbow flexion was 4 (Fig. 1e). Active contraction of the latissimus dorsi muscle flap was confirmed during flexion of the elbow joint under both supination and forearm pronation (Fig. 1f, g). Moreover, flexion strength was measured using a Micro FET^®^ 2 (Hoggan Scientific, Salt Lake City, UT, USA). There was no difference between supination strength at 3.6 kg and pronation strength at 3.5 kg (measurements from the uninjured side were 6.2 kg for supination and 6.0 kg for pronation). Finally, the patient was able to bring the elbow joint from an extended position to 90° flexion with a 4.5 kg weight wrapped around the forearm. Two years after surgery, he returned to his job as an event producer.

### Case 2

**Fig. 2:**
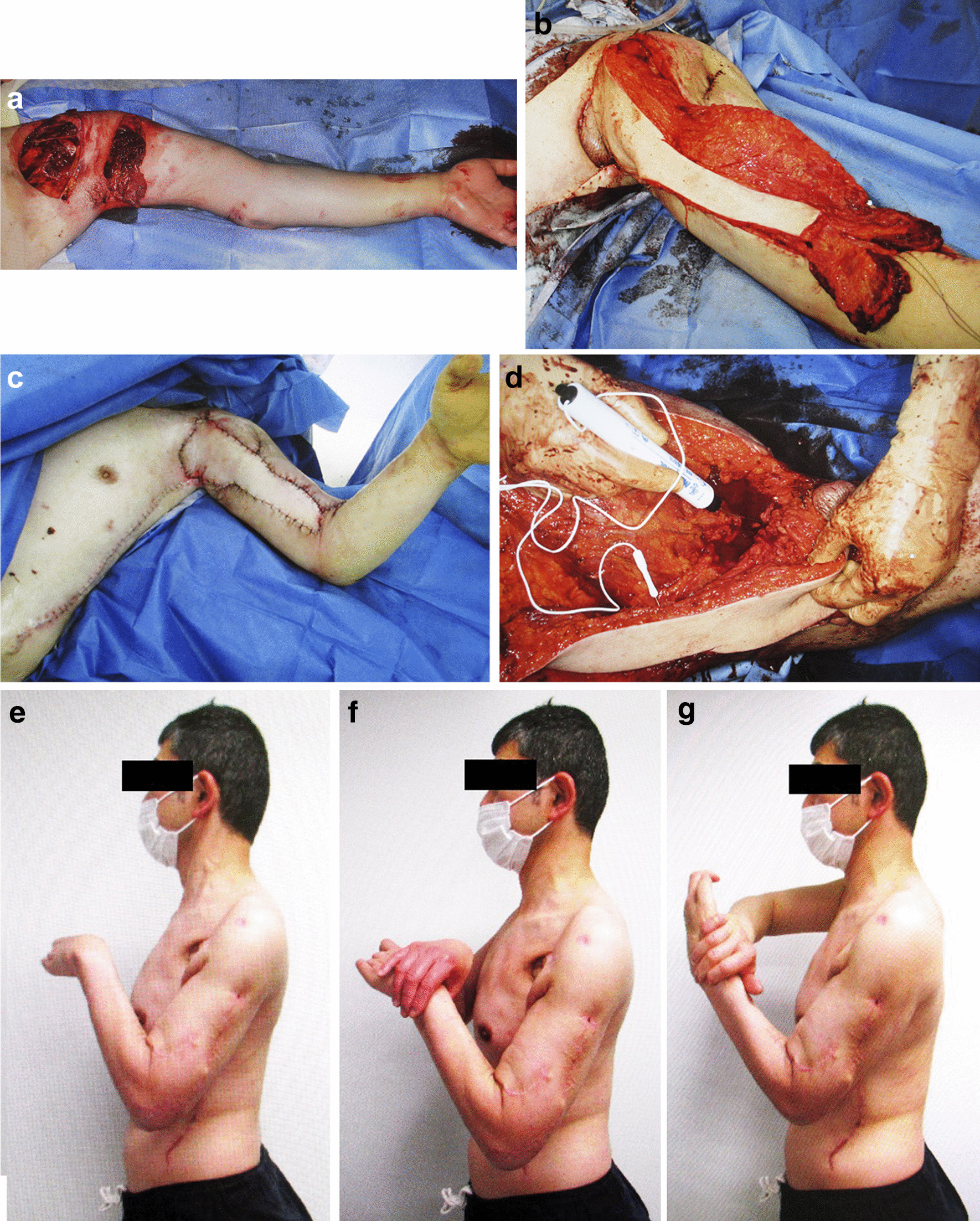
Case 2. **a** Functions of the musculocutaneous nerve and biceps brachii were partially lost due to debridement. **b** The latissimus dorsi muscle flap was stretched to its original length and divided into two (Black squares indicate flaps). **c** The site of soft tissue loss was covered by the transferred latissimus dorsi musculocutaneous flap. **d** Latissimus dorsi muscle contraction was confirmed using electrical stimulation of the thoracodorsal nerve. **e** Elbow joint active range of motion was 20–130°. **f** Forearm pronation with extension resistance. Active contraction of the latissimus dorsi muscle during flexion was confirmed. **g** Active contraction of the latissimus dorsi muscle during flexion was confirmed with forearm supination

Case 2 involved a 50-year-old Asian man who presented with an open wound on the left proximal upper arm after his upper limb was pulled into a conveyor belt. He sustained a deep laceration in the proximal upper arm, resulting in the rupture of the musculocutaneous nerve and contusions of the median, radial, and ulnar nerves. Furthermore, ruptures of the biceps brachii and pectoralis major, fractures of the humerus and transverse process of one cervical vertebra, carpometacarpal dislocation and fracture, and severing of the middle and ring fingers were noted (Fig. 2a). The patient was diagnosed with paralysis of the left biceps brachii and brachialis due to the musculocutaneous nerve rupture and the median, radial, and ulnar nerve contusion. Notably, musculocutaneous nerve injury resulted in the inability to perform elbow flexion. Debridement was performed on the day of injury. Moreover, debridement was performed repeatedly because of soil contamination, resulting in partial loss of the musculocutaneous nerve, biceps brachii, and pectoralis major. Three weeks after the injury was sustained, a bipolar latissimus dorsi muscle with a skin paddle, in which the muscle flap was separated into two and fixed to the radial and ulnar tuberosities, was used to restore elbow joint flexion and reconstruct the soft tissue (Fig. 2b, c). A nerve stimulator was used to confirm the intraoperative contraction of the latissimus dorsi muscle (Fig. 2d). Furthermore, complications of brachial plexus paralysis were identified postoperatively, and the elbow joint flexion strength following latissimus dorsi muscle surgery was assigned an MRC grade of 0. Physiotherapy was initiated during the seventh postoperative week, and the patient began working solely on passive flexion of the elbow joint because of brachial plexus paralysis. Seven months after surgery, the active range of the elbow recovered to 40–105°. Notably, one year after the surgery, the radial and ulnar nerves remained partially paralyzed, with an active range of motion of the elbow between 20–130°, and flexion was assigned an MRC grade of M4 (Fig. 2e). Active contraction of the latissimus dorsi muscle flap was confirmed with forearm supination and pronation during elbow joint flexion (Fig. 2f, g). Measurement with Micro FET^®^ 2 revealed no significant difference between supination at 4.8 kg and pronation at 4.6 kg (measurements from the uninjured side were 7.0 kg for supination and 6.8 kg for pronation), and the patient was able to bring the elbow joint from an extended position to 90° flexion with a 4.0 kg weight wrapped around the forearm. One year after the surgery, he returned to his job as a machine operator.

## Discussion

This report describes two cases of biceps brachii and brachialis paralysis due to musculocutaneous nerve injury, in which bipolar rotational transfer of the latissimus dorsi muscle was used to restore elbow flexion. Notably, a nonfunctioning hand can be used in a stable state if the elbow is capable of flexion [11]. As the elbow joint was flexed from 0° to 60°, the axial load became more significant in the capitellum and trochlea of the humerus [15], implying that both the humeroradial and humeroulnar joints require flexion strength at the elbow joint between 0° and 60° of movement. Furthermore, the brachialis muscle dynamically stabilizes the humeroulnar joint [2] and maintains elbow stability during concentric and eccentric contraction [16]. Thus, flexion requires the isotonic traction of the biceps brachii and brachialis muscles.

In treating distal biceps brachii tendon ruptures, there have been previous discussions regarding methods of anatomical suturing to the radial tuberosity and non-anatomical suturing to the brachialis [17–20]. Non-anatomic repair has also yielded favorable results, restoring 60–96% of flexion strength [18, 20]. Based on these reports, we hypothesized that it would have been possible to ensure flexion stability of both the humeroradial and humeroulnar joints in patients with paralysis of the biceps brachii and brachialis by suturing the latissimus dorsi muscle flap to both the radius and ulna. Moreover, we thought it would have been better to suture the radius and ulna to achieve strong elbow flexion without being affected by supination or pronation of the forearm. However, studies on reconstruction via latissimus dorsi muscle rotational transfer have commonly reported using a surgical technique in which the muscle flap is sutured only to the radial tuberosity [11–14]. Moreover, in a previous case where only the musculocutaneous and radial nerves of the brachial plexus were paralyzed, reconstruction was performed with sutures only on the radius [21]. Thus, there is no standard method for elbow flexion reconstruction, which depends on the etiology and diagnosis of each case.

When the biceps brachii and brachialis are paralyzed owing to musculocutaneous nerve injury, the forearm cannot flex the elbow joint in the pronated or supinated position. Thus, reconstruction using the radial tuberosity alone when there is also brachialis paralysis can be expected to yield poorer flexion strength when the forearm is pronated than when it is supinated. We hypothesized that a latissimus dorsi muscle flap could be sutured to both the radius and ulna to achieve flexion stability in both the humeroradial and humeroulnar joints. However, to the best of our knowledge, no previous report has mentioned this in the literature. The two cases in which this procedure was performed demonstrated that the flexion force in the supinated position was equal to that in the pronated position.

Our reconstruction method used the rotational transfer of the latissimus dorsi muscle. Close attention was paid to the following four points during the surgical procedure.

The first was the preoperative evaluation of whether the inability of the elbow joint to flex also impacted the latissimus dorsi muscle and caused paralysis, as the strength of the latissimus dorsi muscle is directly related to postoperative flexion strength [13]. Notably, several methods are available for evaluating the presence of latissimus dorsi muscle paralysis. For our patients, shoulder adduction with resistance could not be used for evaluation as both had open wounds, and the patient in Case 2 had broken bones. Furthermore, it is difficult to distinguish the motion of the latissimus dorsi from that of the teres major during shoulder adduction [14, 22], and the evaluation of latissimus dorsi muscle contraction upon coughing [11, 13, 14] was not possible in these cases, as neither patient was able to cough forcefully. Some reports have proposed preoperative nerve conduction studies [23, 24]; however, such evaluations are not possible in cases of open wounds or bone fractures due to acute traumatic injury. Instead, we opted for a nerve stimulator to apply electrical stimulation to the thoracodorsal nerve and intraoperatively confirm the muscle contraction. However, in Case 2, brachial plexus paralysis was not detected because it was central to the thoracodorsal nerve. In cases involving fractures of the vertebral transverse process, it is necessary to check for the complications of brachial plexus paralysis.

**Fig. 3:**
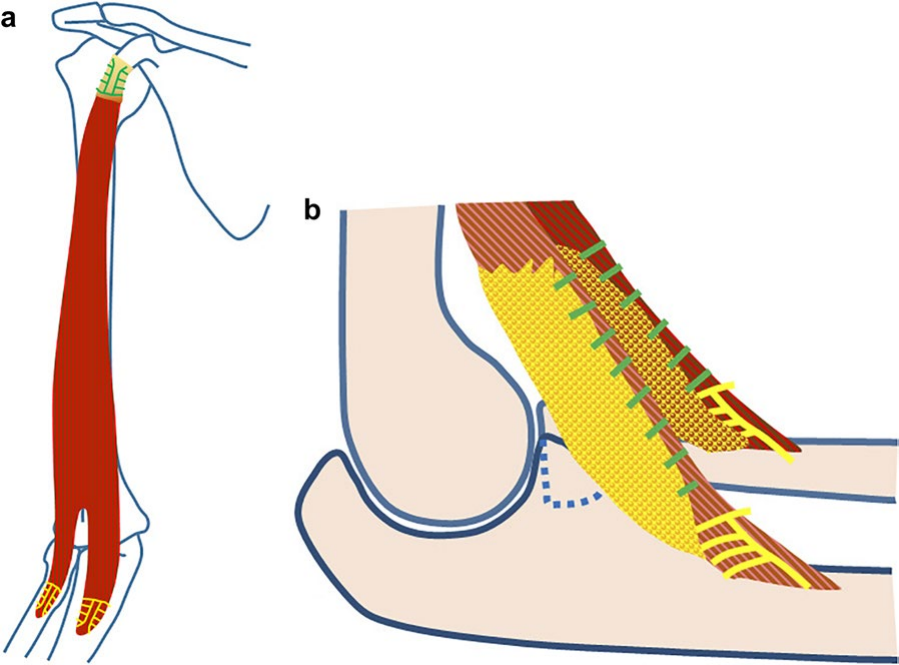
The latissimus dorsi muscle flap is shown here. **a** The transferred latissimus dorsi muscle flap was fixed to the coracoid process, radial and ulnar tuberosities with Krakow sutures. **b** The latissimus dorsi muscle flap was divided into two; the radial side was attached to an anchor on the radial tuberosity, wrapped around the myotendinous junction of the biceps brachii, and the ulnar side was fixed to the ulnar tuberosity, wrapped around the myotendinous junction of the brachialis

The second point on which we focused was the method of latissimus dorsi muscle reconstruction. Lower flexion strength has been reported when the latissimus dorsi muscle is sutured laterally beyond the radial tuberosity [25]. In our study, we excised the nonviable muscle, leaving only the myotendinous junction of the biceps brachii. Specifically, the distal flap of the latissimus dorsi muscle was divided into the radial and ulnar sides. The radial side was attached to an anchor thread that had been driven into the radial tuberosity using Krakow sutures, wrapped around the myotendinous junction of the biceps brachii, and secured with sutures in anatomical position. The superficial head of the brachialis muscle was inserted by a thick circular tendon, and the deep head was inserted into the ulnar tuberosity via the aponeurosis [2]. Therefore, the ulnar side of the muscle flap was fixed proximal or distal to the brachialis muscle, using Krakow-sutured anchors, wrapped around the myotendinous junction of the brachialis, and secured with sutures (Fig. 3a, b). This resulted in equal elbow joint flexion strength in both cases, regardless of whether the forearm was supinated or pronated.

The third point to which we paid close attention was the avoidance of suturing the latissimus dorsi muscle while it was contracted. Reduced flexion strength has been reported when the muscle is not restored to its original length or when the suture thread between the biceps brachii tendon and muscle belly of the latissimus dorsi becomes slack because of the lack of a tendon stump on the distal side of the latissimus dorsi muscle [13]. In our surgical procedures, 5 cm intervals were marked on the latissimus dorsi muscle before dissection. Following the dissection of the proximal musculocutaneous flap from the humerus, the muscle was stretched to restore its length until the interval was 5 cm. Moreover, bipolar transfers to the coracoid, radius, and ulna were performed with the muscle at full length, and the coracoid suture site was adjusted to ensure tension, allowing the elbow joint to remain at a 90° flexion angle after fixation. Notably, a previous report described a surgical technique to increase the flexion strength through fixation of the latissimus dorsi muscle to the proximal one-third of the ulnar diaphysis to increase the lever arm length [22]. However, in these cases, we achieved a satisfactory range of motion and strength by restoring the muscle flap length and wrapping around the myotendinous junction of the biceps brachii and brachialis before securing it with sutures, enabling flexion of both the humeroradial and humeroulnar joints.

Fourth, we focused on using musculocutaneous flaps rather than muscle flaps. This allowed flap monitoring and improved the muscle and tendon gliding of the transferred latissimus muscle [14]. In this study, we did not use a subcutaneous tunnel. Instead, we extended the open wound with an incision and transferred the latissimus dorsi musculocutaneous flaps so that the skin paddle fit the space, simultaneously restoring elbow flexion and reconstructing the soft tissue. As there was no separate tissue between the skin and muscle of the latissimus dorsi, adhesions did not occur, and we successfully confirmed the active contraction of the latissimus dorsi muscle with the forearm in both the supination and pronation positions. Notably, distal tip necrosis of the flap has been previously reported when a pedicled latissimus dorsi flap is used to cover a defect around the elbow [3, 26]. Therefore, the musculocutaneous flaps should be carefully monitored.

For these surgeries, we sutured the latissimus dorsi muscle at the myotendinous junction of the biceps brachii and brachialis, as we had sufficient margin to split the muscle flap. By suturing both pieces, we achieved stability of the humeroradial and humeroulnar joints and elbow flexion strength, which were not affected by the rotational position of the forearm.

As this is a case report, it is necessary to conduct further research to verify the effectiveness of this new fixation method. Traumatic injuries, such as those in the present cases, where musculocutaneous nerve suture and grafting are not feasible, are rare; therefore, the inability to quickly accumulate cases is a disadvantage. In addition, because the strength of the latissimus dorsi muscle becomes equal to the elbow joint flexion strength, it is often impossible to use this surgical method in cases of brachial plexus paralysis.

## Conclusion

This study reported two cases of biceps brachii and brachialis paralysis due to musculocutaneous nerve injury, wherein we reconstructed elbow joint flexion using latissimus dorsi muscle rotational transfer with sutures to the radial tuberosity and ulnar tuberosity, thereby enabling flexion by simultaneous activation of the humeroradial and humeroulnar joints. We achieved stability in the humeroradial and humeroulnar joints and restored adequate range of motion and flexion strength in the elbow joint, confirming that the flexion force in the supinated position was equal to that in the pronated position.

## Data Availability

The dataset used and analyzed during the current study is available from the corresponding author on reasonable request.

## Abbreviation

MRC: British Medical Research Council grade
